# Disturbances in the ocular surface microbiome by perioperative antimicrobial eye drops

**DOI:** 10.3389/fcimb.2023.1172345

**Published:** 2023-04-12

**Authors:** Fumika Hotta, Hiroshi Eguchi, Tomomi Kuwahara, Haruyuki Nakayama-Imaohji, Yoshikazu Shimomura, Shunji Kusaka

**Affiliations:** ^1^ Department of Ophthalmology, Kindai University Faculty of Medicine, Osaka, Japan; ^2^ Department of Molecular Microbiology, Kagawa University Faculty of Medicine, Kagawa, Japan; ^3^ Department of Ophthalmology, Fuchu Eye Center, Osaka, Japan

**Keywords:** ocular surface, microbiome, diversity, perioperative antimicrobial eye drops, 16S rRNA

## Abstract

We aimed to elucidate the effects of antimicrobial eye drops used in the perioperative period of ophthalmic surgery on the ocular surface microbiome by metagenomic analysis. Twenty-eight eyes from 15 patients (mean age 74.1 years) with no history of eye drop use within 3 months before cataract surgery were included in this study. Gatifloxacin eye drops were used in all patients in the perioperative period. The antimicrobial eye drops were started 3 days before surgery. They were discontinued after conjunctival sac specimen collection for 2 weeks after the surgery. Conjunctival sac specimens were collected to investigate the alterations in the ocular surface microbiome by meta-16S analysis targeting the V3-V4 region of the bacterial 16S rRNA gene. Principal coordinate analysis showed that the bacterial composition tended to be different before and 2 and 4 weeks after surgery. Individual observations on six eyes showed that the bacterial composition at 12 weeks after surgery was closer to that before surgery than to that at 4 weeks after surgery in two eyes, while the bacterial composition in the remaining four eyes was different at various time points. Before surgery, Firmicutes, Proteobacteria, and Bacteroidetes were predominant; however, 2 weeks after surgery, the proportion of Proteobacteria increased and that of Firmicutes decreased. A similar trend was noticed 4 weeks after surgery, although antibacterial eye drops had been discontinued 2 weeks after surgery. The Shannon–Weaver coefficient showed a decreasing trend at 2-, 4-, and 12-weeks post operation compared to that before operation. The diversity of the microbiome decreased significantly at 2- and 4-weeks after surgery when compared to that before surgery (p < 0.05). The ocular surface microbiome is easily disrupted by antimicrobial eye drops, and it needs recovery time. In such cases, the ocular surface microbiome is presumed to contain many antimicrobial-resistant bacteria. In some cases, it may not recover, and a new microbiome is formed.

## 1 Introduction

Acute bacterial endophthalmitis after intraocular surgery is a serious complication and can lead to blindness. Although the incidence following cataract surgery is low, the absolute number of cases is high owing to an enormous number of surgeries, thereby making it a concerning health issue worldwide (Gower et al., 2017). Various efforts have been made to avoid acute bacterial endophthalmitis. The prophylaxis regimen in the perioperative period is useful but uncertain. Meta-analyses have revealed intracameral injection of cefuroxime at the end of surgery as the most effective prophylaxis (ESCRS Endophthalmitis Study Group, 2007), followed by intraoperative cefuroxime or penicillin injection and postoperative levofloxacin (LVFX) or chloramphenicol eye drops with moderate certainty (Gower et al., 2017). However, the administration of preoperative antibiotic eye drop is questionable (Ciulla et al., 2002; Kessel et al., 2015), and no global consensus has ascertained their preventive effects. The administration of 0.5% LVFX eye drops has been recommended four times a day starting 3 days before surgery because the number of bacteria isolated from the ocular surface immediately before surgery is the lowest (Inoue et al., 2008). The reduction of viable bacteria on the ocular surface prior to an intraocular surgery is reasonable because most causative bacteria for postoperative endophthalmitis originate from the ocular surface microbiome. Therefore, reducing the number of viable bacteria on the ocular surface with quinolone eye drops for 3 days before surgery, administering cefuroxime in the anterior chamber at the end of surgery, and using quinolone eye drops for several days after surgery may be effective in preventing the development of acute bacterial endophthalmitis. However, to the best of our knowledge, there is no clear recommendation on when to discontinue postoperative use of antimicrobial eye drops. In one report, LVFX has been administered for 6 days after surgery (ESCRS Endophthalmitis Study Group, 2007), although specific reasons behind its discontinuation was not mentioned. Antimicrobial eye drops are probably discontinued worldwide based on an empirical judgment of institutions or surgeons; however, they are sometimes administered unnecessarily for a long period. Prolonged use of antimicrobial eye drops should be avoided because it leads to an induction of resistance in commensal bacteria and the development of an abnormal ocular surface microbiome dominated by antimicrobial resistant-bacteria owing to the selection pressure. Therefore, the duration of postoperative use of antimicrobial eye drops needs to be validated.

In recent years, an increase in microbiome analysis using next-generation sequencing (NGS) technologies in ophthalmology has been observed (Dong et al., 2011; Zhou et al., 2014; Huang et al., 2016; Eguchi et al., 2017; Ozkan et al., 2017; Hotta et al., 2020; Zilliox et al., 2020). Some authors (Dong et al., 2011; Eguchi et al., 2017) have reported the presence of gram-negative rods in the microbiome, which are presumed to be of environmental origin, unlike the results of ocular surface cultures reported earlier. DNA sequencing experiments contain information on non-viable bacteria; therefore, the gram-negative rods detected may not be the causative agents of infectious diseases. Differences in specimen collection methods can also greatly affect the results, since NGS is a highly sensitive test and the ocular surface contains a small number of microorganisms, unlike the gut. If the results are not properly normalized, the actual ocular surface microbiome may not be well-elucidated (Ozkan et al., 2017). Therefore, standardized methods of specimen collection and analysis may offer a clear view on the relationship between disease or pathophysiology and changes in the ocular surface microbiome, which would facilitate the understanding and management of clinical course of diseases. In this study, we aimed to determine the impact of perioperative antimicrobial eye drops on the ocular surface microbiome using NGS technologies and to propose an appropriate time to discontinue eye drops after surgery.

## 2 Methods

### 2.1 Participants

Twenty-eight eyes from 15 patients (10 eyes from 5 men and 18 eyes form 10 women) with a mean age of 74.1 years (49–88 years) with no history of ophthalmic drug use, including over-the-counter drugs, within 3 months before cataract surgery were included. The present study followed the tenets of the Declaration of Helsinki and was approved by the Institutional Review Board of Kindai University Faculty of Medicine (approval no. 28-137). Informed consent was obtained from all subjects after explaining the nature and possible consequences of the study.

### 2.2 Sample collection

All patients received antimicrobial eye drops four times daily. Ocular surface specimens were collected approximately 3−4 hours after the first round of drop. One milliliter of physiological saline, which washed the ocular surface, was collected with a sterile pipette as the sample containing the ocular surface microbiome. Different pipettes and aliquots of saline were used for each eye. Samples were collected before surgery and 2-, 4-, and 12-weeks after surgery. In all patients, gatifloxacin (Gatiflo^®^ ophthalmic solution 0.3%; GFLX), betamethasone sodium phosphate (Sanbetason^®^ ophthalmic and otorhinologic solution 0.1%), and bromfenac sodium (Bronuck^®^ ophthalmic solution 0.1%) eye drops were used perioperatively. GFLX eye drop was started 3 days before surgery and was discontinued after 2 weeks of surgery. Betamethasone phosphate and bromfenac sodium ophthalmic solutions were discontinued after 4 weeks of surgery.

### 2.3 Microbiome analysis

Samples were centrifuged (16,000 × *g* for 5 min at 4°C) within 10 min of collection. DNA was extracted as described previously (Morita et al., 2007). DNA was amplified by REPLI-g Single Cell Kit (cat. no. 150345, QIAGEN) according to the manufacturer’s instructions. Sequencing libraries were prepared by amplifying the V3-V4 region of the 16S rRNA gene using the primers (5′−TCG TCG GCA GCG TCA GAT GTG TAT AAG AGA CAG CCT ACG GGN GGC WGC AG−3′ and 5′−GTC TCG TGG GCT CGG AGA TGT GTA TAA GAG ACA GGA CTA CHV GGG TAT CTA ATC C−3′) as previously reported (Hotta et al., 2020). Sequencing was performed using Illumina MiSeq platform (cat. no. MS-102-3003, MiSeq Reagent Kit ver. 3, 600 cycles, Illumina, Inc.) according to the manufacturer’s instructions, to generate paired-end reads of 300 bases in length in each direction. The same saline solution for sample collection was used as negative control and analyzed using the same reagents and methods for sample analysis. To eliminate the effect of contamination during experiment, the operational taxonomic units (OTUs) in the negative control were subtracted from that in the specimen in the case of same OTUs in specimens and negative control. Using the QIIME 2 pipeline (https://view.qiime2.org/), weighted UniFrac distances were generated and used to investigate beta diversity through principal coordinate analysis (PCoA), create a bar chart of relative abundance, and alpha diversity indices between groups were compared using the Kruskal–Wallis test.

## 3 Results

### 3.1 Principal coordinate analysis

**Figure 1:**
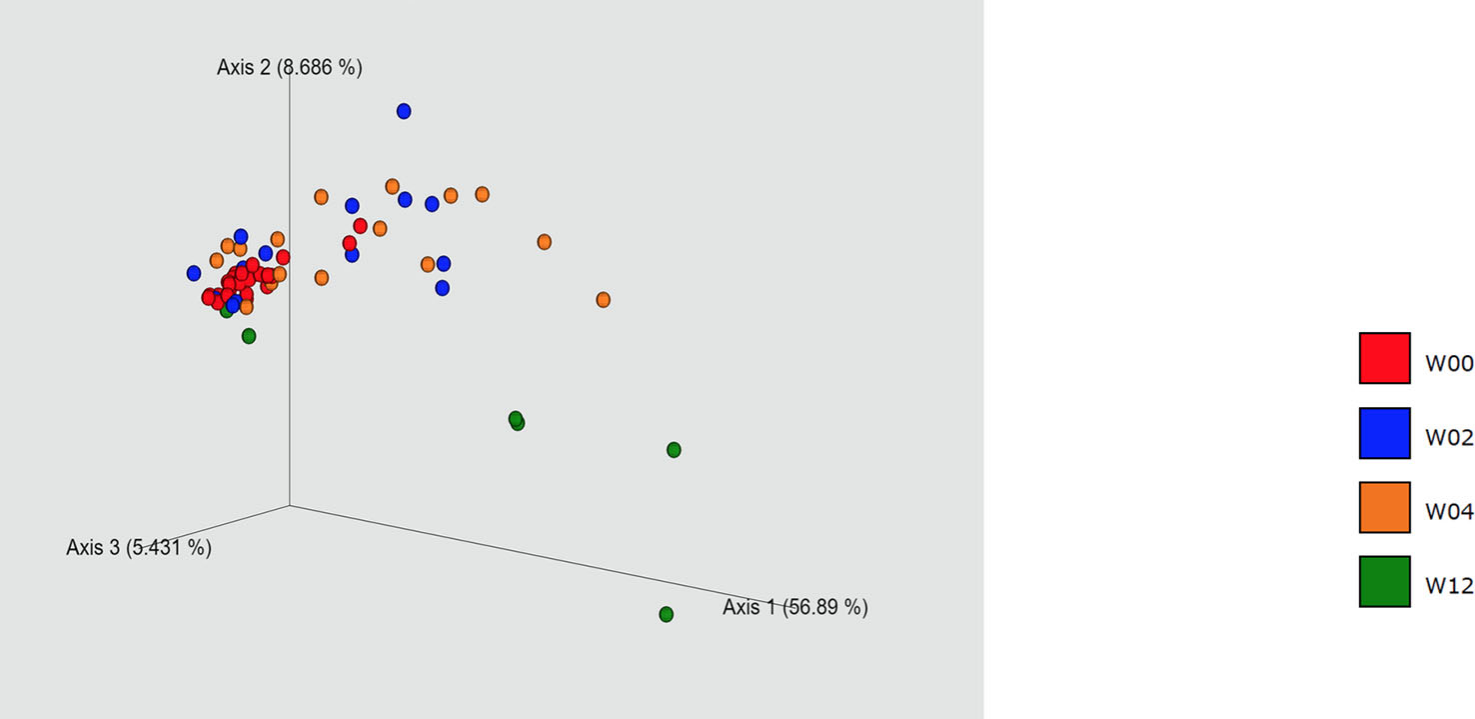
Principal coordinate analysis. W00, W02, W04, and W12 represent before operation and 2-, 4-, and 12 -weeks after operation, respectively. Some of the W12 specimens showed a composition close to that of the preoperative one, while the others showed a considerable difference.

Of the 28 eyes in 15 cases, specimens were collected preoperatively (W00), and 2- (W02), 4- (W04), and 12-weeks (W12) after operation from 28, 14, 16, and 6 eyes, respectively. PCoA showed different bacterial composition in W00, W02, and W4. In particular, a chronological comparison of bacterial composition, of the three cases and six eyes that had been followed from W00 to W12, showed that the bacterial composition at W12 was closer to that at W00 than W04 in one case and two eyes. Conversely, in four eyes of two cases, the bacterial composition at W12 differed significantly from that at W00 (**Figure 1**).

### 3.2 Relative abundance

**Figure 2:**
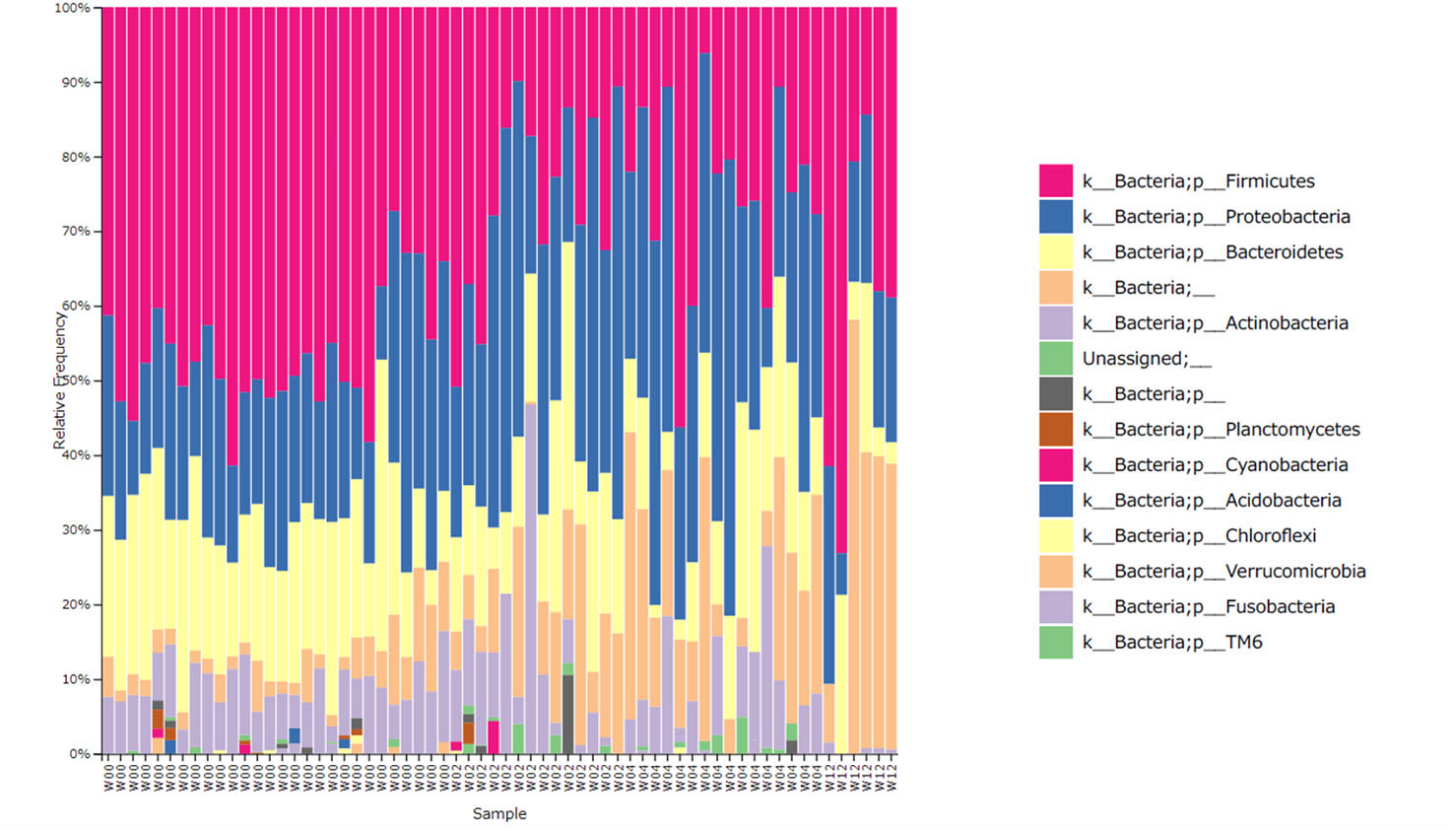
Relative abundance of the bacterial phylum level. W00, W02, W04, and W12 represent before operation and 2-, 4-, and 12-weeks after operation, respectively. The proportion of Firmicutes decreased and that of Proteobacteria increased with time. At 12 weeks post operation, some specimens showed a recovered proportion of Firmicutes.

**Figure 2** shows the bacterial composition at the phylum level, according to the time of collection. In W00, Firmicutes, Proteobacteria, and Bacteroidetes were predominant, but in W02, the proportion of Proteobacteria increased and Firmicutes decreased. The same trend in bacterial composition was observed in W04 although antimicrobial eye drops were discontinued after W02.

### 3.3 Shannon–Weaver index

**Figure 3:**
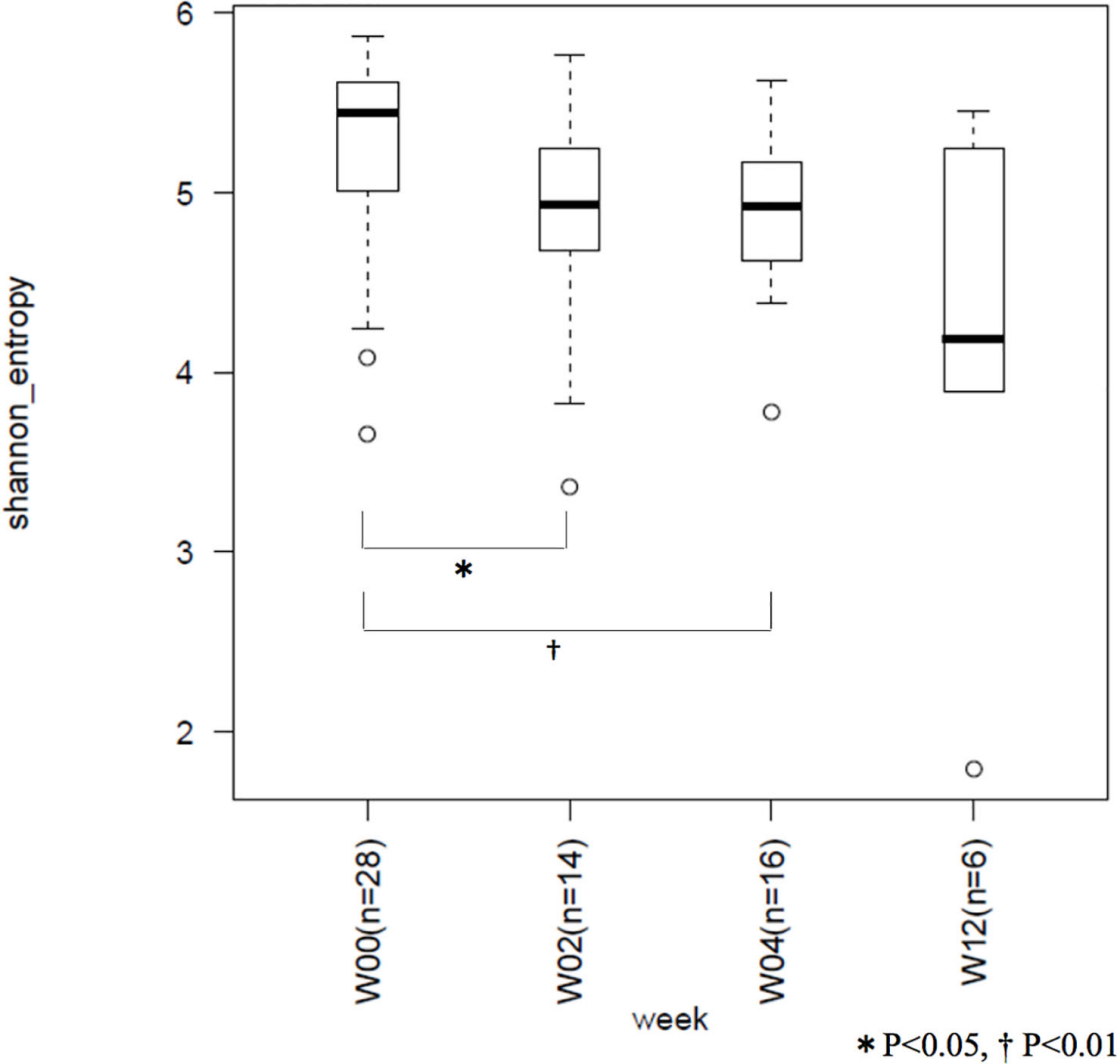
Between-group comparison of Shannon coefficients. W00, W02, W04, and W12 represent before operation and 2-, 4-, and 12-weeks after operation, respectively. Microbiome diversity showed a decrease with time.

The mean Shannon coefficients for W00, W02, W04, and W12 were 5.24 ± 0.57, 4.81 ± 0.70, 4.87 ± 0.47, and 4.13 ± 1.31, respectively. Compared to the preoperative period, there was a significant decrease in the index at 2- (*p* = 0.037) and 4- (*p* = 0.006) weeks post operation. Average of the index did not increased at W12. No significant difference was observed between the indices of 2, 4, and 12 weeks post operation (**Figure 3**).

## 4 Discussion

Perioperative bacterial reduction of the ocular surface is aimed to prevent acute bacterial endophthalmitis after cataract surgery; however, the selection pressure exerted by antimicrobial substances reduces the diversity of the normal flora, which is detrimental to organ homeostasis. *Staphylococcus epidermidis* has been frequently isolated as the causative bacteria of post-cataract surgery endophthalmitis (Bannerman et al., 1997; Das et al., 2003; Jeong et al., 2017), which indicates the possibility of specimen contamination. Alternatively, the number of endophthalmitis caused by quinolone and/or methicillin-resistant *Staphylococcus* (Deramo et al., 2008; Slean et al., 2017) may increase owing to the selection pressure exerted by quinolone on the ocular surface microbiome.

A scientific rationale is necessary to shorten the postoperative administration period of quinolone eye drops since it may induce the development of endophthalmitis. However, a multicenter prospective study is practically difficult to compare the incidence of endophthalmitis in a group using antimicrobial eye drops for a certain period after surgery (e.g., more than 1 month) and another group using them for a shorter period (e.g., less than 2 weeks). A multicenter prospective study on the development of post-cataract surgery endophthalmitis in more than 100,000 candidates failed to find significant risk factors because of the low (approximately 0.02%) incidence of endophthalmitis (Inoue et al., 2018). A comparison between the two aforementioned groups is not expected to yield statistically significant results. Therefore, exploring the appropriate time to discontinue antimicrobial eye drops post operation based on accumulated results of basic studies such as the present study is a practical approach.

The use of LVFX eye drops for 2 weeks after cataract surgery increased the induction of quinolone resistance in bacteria isolated from the ocular surface (Miyanaga et al., 2009); 63% of coagulase-negative *Staphylococcus* isolated from the ocular surface before surgery were susceptible to LVFX, which drastically decreased to 12%, and 69% of isolated strains showed resistance to LVFX following the use of LVFX eye drops for 2 weeks after surgery. The total number of isolates decreased after 2 weeks of LVFX administration and the drastic increase in the frequency of resistant isolates indicated that LVFX-susceptible strains drastically decreased because of the selection pressure. This result indicated that the disturbed ocular surface microbiome, containing mainly the resistant strains, was also detected by conventional culture. Similar to the results published by Miyanaga et al., our metagenome analysis also demonstrated a decrease in the relative abundance of Firmicutes, which contain Gram-positive bacteria such as *Staphylococcus* sp., after administering the GFLX eye drops for 2 weeks.

We found that the downward trend of the ocular surface microbiome diversity persisted even after discontinuation of antimicrobial eye drops. This may be because of a stressful selection pressure of broad-spectrum antimicrobial eye drops used for 2 weeks. The amount of bacteria in the ocular surface is originally low; therefore, their recovery from antimicrobial stress may be difficult. Two of the 6 eyes that were followed up to 12 weeks postoperatively showed a similar microbiome composition before surgery. Therefore, some of the microbiomes can return to their original state after three or more months post operation. However, the remaining four eyes had ocular surface microbiomes different from their preoperative ones. It may acquire a new stable microbiome as a microbial community undergoes a dynamic process to evolve toward another stable state in response to an external perturbation (He et al., 2022). Alternatively, the ocular surface microbiome may need more than 3 months to recover after the administration of quinolone eye drop only for 2 weeks. Quinolone eye drops should be discontinued within two weeks after successful cataract surgery. It may be better to discontinue earlier, for example, one week after surgery. Long-term analyses of a large number of cases may provide new insights into the resilience of the ocular surface microbiome.

The current study had some limitations. First, the sample size was small as the study population consisted of candidates who had not used any eye drops, including over-the-counter one, for at least 3 months. Second, we adopted the results for both eyes in cases where simultaneous surgery was performed on them, although the information of only one eye in one patient should be analyzed. As both eyes may not have a similar microbiome, we considered both eyes for examination. Third, antibacterial eye drops were discontinued 2 weeks after surgery; however, steroid and nonsteroidal anti-inflammatory drugs were continued as eye drops. The effect of preservatives that is present in these eye drops on the ocular surface microbiome cannot be excluded. The impact of the microbiome changes identified in this study on ocular physiology remains unclear. However, it is necessary to accumulate data on the correlation between the ocular surface microbiome changes after antimicrobial eye drop use, postoperative outcomes, and isolation rates of resistant bacteria on the ocular surface in order to understand the role of the true ocular surface microbiome and its changes after antimicrobial eye drop administration in the physiological functions of the eye.

In conclusion, the use of antimicrobial eye drops post operation may lead to a reduction in the diversity of the ocular surface microbiome and result in the formation of new microbiome. Therefore, ophthalmologists should refrain from prescribing antimicrobial eye drops for an unnecessarily long perioperative period.

## Data availability statement

The datasets presented in this study can be found in online repositories. The names of the repository/repositories and accession number(s) can be found below: https://www.ddbj.nig.ac.jp/, DRA015688.

## Ethics statement

The studies involving human participants were reviewed and approved by Institutional Review Board of Kindai University Faculty of Medicine (approval no. 28 137). The patients/participants provided their written informed consent to participate in this study.

## Author contributions

FH, HE, and TK contributed to conception and design of the study. FH and HE collected samples. FH and HNI organized the database. FH, TK, and HNI performed the statistical analysis. FH and HE wrote the first draft of the manuscript. YS and SK reviewed the manuscript. All authors contributed to manuscript revision, read, and approved the submitted version.
